# Novel risk models for early detection and screening of ovarian cancer

**DOI:** 10.18632/oncotarget.13648

**Published:** 2016-11-26

**Authors:** Matthew R. Russell, Alfonsina D'Amato, Ciaren Graham, Emma J Crosbie, Aleksandra Gentry-Maharaj, Andy Ryan, Jatinderpal K. Kalsi, Evangelia-Ourania Fourkala, Caroline Dive, Michael Walker, Anthony D. Whetton, Usha Menon, Ian Jacobs, Robert L.J. Graham

**Affiliations:** ^1^ Stoller Biomarker Discovery Centre and Pathology Node, Division of Molecular and Clinical Cancer Sciences, Faculty of Biology, Medicine and Health, University of Manchester, Manchester, UK; ^2^ School of Healthcare Science, Manchester Metropolitan University, UK; ^3^ Gynaecological Oncology Research Group, Division of Molecular and Clinical Cancer Sciences, Faculty of Biology, Medicine and Health, University of Manchester, Manchester, UK; ^4^ Gynaecological Cancer Research Centre, Women's Cancer, Institute for Women's Health, University College London, London, UK; ^5^ Clinical and Experimental Pharmacology Group, Cancer Research UK Manchester Institute, University of Manchester, Manchester, UK; ^6^ University of New South Wales, Australia

**Keywords:** ovarian cancer, UKCTOCS, early detection, logit, risk estimation

## Abstract

**Purpose:**

Ovarian cancer (OC) is the most lethal gynaecological cancer. Early detection is required to improve patient survival. Risk estimation models were constructed for Type I (Model I) and Type II (Model II) OC from analysis of Protein Z, Fibronectin, C-reactive protein and CA125 levels in prospectively collected samples from the United Kingdom Collaborative Trial of Ovarian Cancer Screening (UKCTOCS).

**Results:**

Model I identifies cancers earlier than CA125 alone, with a potential lead time of 3-4 years. Model II detects a number of high grade serous cancers at an earlier stage (Stage I/II) than CA125 alone, with a potential lead time of 2-3 years and assigns high risk to patients that the ROCA Algorithm classified as normal.

**Materials and Methods:**

This nested case control study included 418 individual serum samples serially collected from 49 OC cases and 31 controls up to six years pre-diagnosis. Discriminatory logit models were built combining the ELISA results for candidate proteins with CA125 levels.

**Conclusions:**

These models have encouraging sensitivities for detecting pre-clinical ovarian cancer, demonstrating improved sensitivity compared to CA125 alone. In addition we demonstrate how the models improve on ROCA for some cases and outline their potential future use as clinical tools.

## INTRODUCTION

The estimation of cancer risk has the potential to improve patient survival through earlier diagnosis and treatment. This approach may prove especially beneficial in ovarian cancer (OC) which is largely asymptomatic in the early stages. OC can be classified as either Type I or Type II. Type I OC includes low grade serous, endometrioid, clear cell, and mucinous carcinomas which are generally indolent, relatively genetically stable and lack TP53 mutations. Type II OC includes high-grade serous and endometrioid, undifferentiated carcinomas and carcinosarcomas that are highly aggressive, evolve rapidly and display TP53 mutations in over 80% of cases [1, 2]. The majority of OC cases are diagnosed at a late stage, resulting in high case fatality. The overall 5 year survival for Stage III & IV is only 23%, making this the most lethal of the gynaecological cancers [3–5]. When detected early prognosis is much better, with approximately 90% of women diagnosed at Stage I surviving five years or more [5].

Therefore, there is a clinical need to develop strategies which can detect OC early. Serum biomarkers are attractive targets for early detection protocols and CA125 has been widely used in screening trials [6–11]. CA125 has limitations of specificity for OC as elevation in serum can also occur in pregnancy, endometriosis and menstruation [12]. Panels of biomarkers are thought to offer the potential for higher discriminatory power. However, recent studies which constructed putative biomarker panels with samples from the Prostate, Lung, Colorectal and Ovarian (PLCO) cancer trial found no improvement in diagnostic power in pre-clinical samples [7, 13] leaving CA125 as the single best biomarker for OC screening. To improve outcomes for OC patients there is a necessity to identify new biomarkers and develop risk models, algorithms and clinical protocols that are capable of improving on or complementing CA125 for early detection of OC [14]. Such biomarkers can only be identified with initial discovery work carried out in prospectively collected sample sets.

This study has developed two risk based models for the early detection of OC. The aim is to assess their utility in comparison to the best current biomarker, CA125, and to see if they can improve on the Risk of Ovarian Cancer Algorithm (ROCA). The serum samples used in this study came from the UKCTOCS cohort of 202,638 women [9, 15, 16]. This sample set contained serial pre-clinical samples for 50,000 of these women providing a unique opportunity for pre-clinical biomarker discovery targeting early stage OC. The models were constructed by analysis of the concentrations of putative biomarkers; Protein Z, and Fibronectin both of which were recently identified as putative biomarkers for ovarian cancer [17]. In conjunction with C-reactive protein, identified as a potential OC marker from the literature [18, 19]. The biomarkers (Protein Z, Fibronectin, C-reactive protein) were combined with CA125, in a nested case-control study within UKCTOCS to determine if a model including four markers would outperform CA125/ROCA-alone. The first, Model I was able to detect Type I whilst the second, Model II was able to detect Type II OCs. The success of the new models to improve over and above CA125 and ROCA are measured through several metrics: the enhanced discrimination between cases and controls, the earlier detection of cases (increased lead time), and in improved sensitivity.

## RESULTS

### Construction of predictive models

The diagnostic potential of the panels of biomarkers were investigated by building separate logistic prediction models (logit models) for Type I (Table 1) and Type II (Table 2) OC cases. When analysing the sensitivity and specificity of the models ROC curves were constructed for investigation over two time ranges 0-1 years tDx and 1-2 years tDx.

**Table 1:** Model I, with coefficients for each variable, the standard error of the estimate and P value

	Coefficient	Standard Error	*P* value	LOO Standard Deviation
(Intercept)	−3.53	1.25	0.005	0.28
CA125	3.74	1.32	0.005	0.35
PROZ	2.90	1.30	0.026	0.31
FINC	−1.50	0.89	0.093	0.31
Age	0.01	0.08	0.948	0.03
PROZ × Age	−0.67	0.22	0.003	0.06
FINC × Age	1.21	0.45	0.007	0.12

**Table 2:** Model II, with coefficients for each variable, the standard error of the estimate and P value

	Coefficient	Standard Error	*P* value	LOO Standard Deviation
(Intercept)	−1.85	0.58	0.001	0.13
CA125	2.53	0.66	0.000	0.13
Age	−0.02	0.09	0.816	0.02
FINC	0.53	0.41	0.197	0.10
CRP	0.59	0.32	0.065	0.05
FINC × Age	0.26	0.11	0.023	0.02

Model I, was trained to identify Type I OC cases. The model (Table 1) included protein expression of CA125, Fibronectin and Protein Z, age of the women at sample collection and interaction terms between each of the proteins: Fibronectin and Protein Z, with age.

Model II, was trained to identify Type II OC cases. The model (Table 2) included protein expression of CA125, Fibronectin and CRP, age of the women at sample collection and interaction terms between Fibronectin with age.

### Validation of models by leave-one-out cross validation

The models were assessed for overfitting using established methodologies for sample sets of this size in ovarian cancer research [20–22] using leave-one-out cross validation. Samples from each subject in turn were excluded from the dataset; the model retrained and logit scores predicted for excluded samples. The root mean square errors between logit scores predicted for samples from models built with them excluded from the model, against the complete model, were 0.024 for Type-I and 0.020 for Type-II demonstrating that the models are not overfitted and are robust to exclusion of subsets of samples.

### Improved descriptive power of models compared to model for CA125 alone

Likelihood ratio tests comparing Model I and Model II with nested logit models constructed for CA125 alone (logit_CA125_) comparing residual variance using the chi square test showed both models fit the data better than CA125 alone (Type-I *P* < 2 × 10^−07^; Type-II *P* = 0.018).

### Epidemiological factors do not add to models

Likelihood ratio tests comparing expanded versions of Model I and Model II, to include additional epidemiological factors of BMI and use of the contraceptive pill or hormone replacement therapy as determined at recruitment, were no better at describing the data (Model I *P* = 0.56 Model II *P* = 0.051). The borderline significant reduction in residual variance in the expanded version of Model II was due to contraceptive pill use. Lower pill use in the OC cases compared to controls in this sample may reflect the known protective effect of the pill, crucially the biomarkers are not correlated with pill use and so not a biological proxy for a readily available epidemiological factor.

### Evaluation of model performance compared to CA125 alone

**Figure 1:**
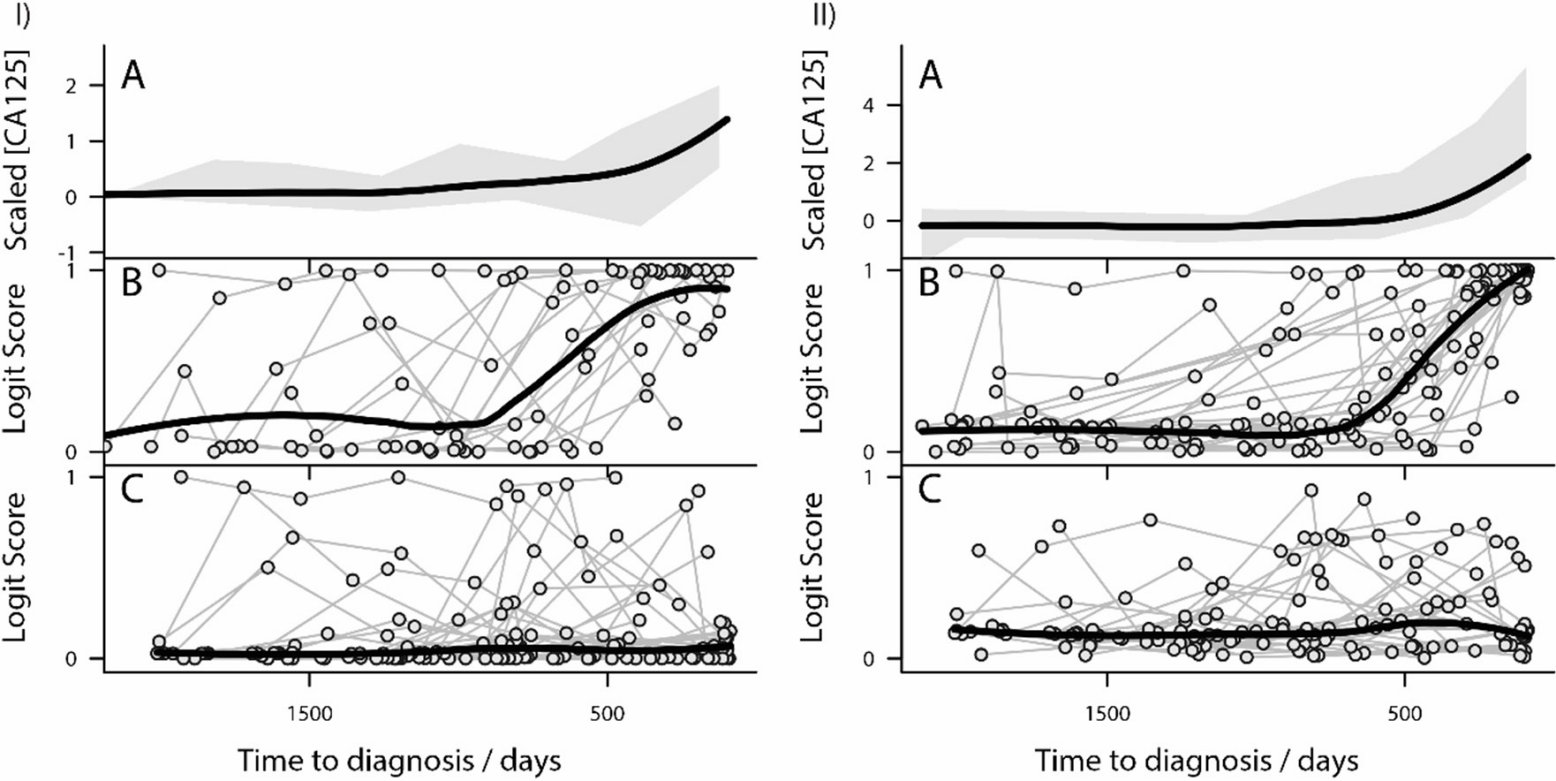
Risk models outperform CA125 for Type I and Type II ovarian cancer (I) Analysis of Type I ovarian cancer patients (**IA**) Loess linear regression analysis of trends in CA125 levels in Type I subjects. The grey shaded areas represent a moving estimate of the 80% percentile range of expression. The thick black line represents the trend in the data identified by the Lowess analysis. (**IB**) Lowess linear regression analysis of the Model I for Type I subjects. Ovarian cancer patients are represented by circles and individual patient levels over time are shown by connected circle. The thick black line represents the trend in the data identified by the Loess analysis. Notice the dramatic and sequential rise in risk estimate as Type I subjects approach diagnosis. (**IC**) Lowess linear regression analysis of Model I for control subjects. Control patients are represented by circles and individual patient levels over time are shown by connected circle. The thick black line represents the trend in the data identified by the Lowess analysis. (II) Analysis of Type II ovarian cancer patients (**IIA**) Lowess linear regression analysis of trends in CA125 levels in Type II subjects. The grey shaded area represents a moving estimate of the 80% percentile range of expression. The thick black line represents the trend in the data identified by the Lowess analysis. (**IIB)** Lowess linear regression analysis of Model II for Type II subjects. Ovarian cancer patients are represented by circles and individual patient levels over time are shown by connected circle. The thick black line represents the trend in the data identified by the Loess analysis. Notice the dramatic and sequential rise in risk estimate as Type II subjects approach diagnosis. (**IIC**) Lowess linear regression analysis of the Model II for control subjects. Control patients are represented by circles and individual patient levels over time are shown by connected circle. The thick black line represents the trend in the data identified by the Lowess analysis.

The serial samples span a timeframe up to six years pre-diagnosis of OC, this enabled us to analyse both time course changes in our Model scores with that of CA125 alone (the best current biomarker) and ROCA for OC cases and to compare Model scores for OC cases with those for controls (Figure 1). Lowess linear regression analysis of these plots show a much more dramatic shift in CA125 velocity within Type II OC subjects (Figure 1 IIA) compared to Type I (Figure 1 IA), whilst the controls show consistently low and flat expression for the respective Model scores across the time course.

In some control samples (Figure 1 IC and IIC) the levels of the markers have shown an increase in risk (a Model score closer to 1), however these levels then decrease towards the normal range in subsequent serial samples. This is consistent with what is seen in the clinical setting. By comparison, in true OC cases (Figure 1 IB and IIB) when an individual demonstrates an elevation in risk, typically this risk continues to increase in subsequent serial samples.

### Increased lead time over CA125 alone

Lowess analysis on the OC cases plots (Figure 1 IB and IIB) demonstrates that for Type I the inflection point in the lowess analysis is about 1000 days (~3 years) time-to-diagnosis (tDx), and for Type II the inflection point in the lowess analysis is ~800 days (~2 years) tDx, these precede the inflection points seen with the CA125 levels alone which occur at about 400 days (~1 year) tDx.

### Model I outperforms CA125 and ROCA

#### Area under the curve analysis

**Figure 2:**
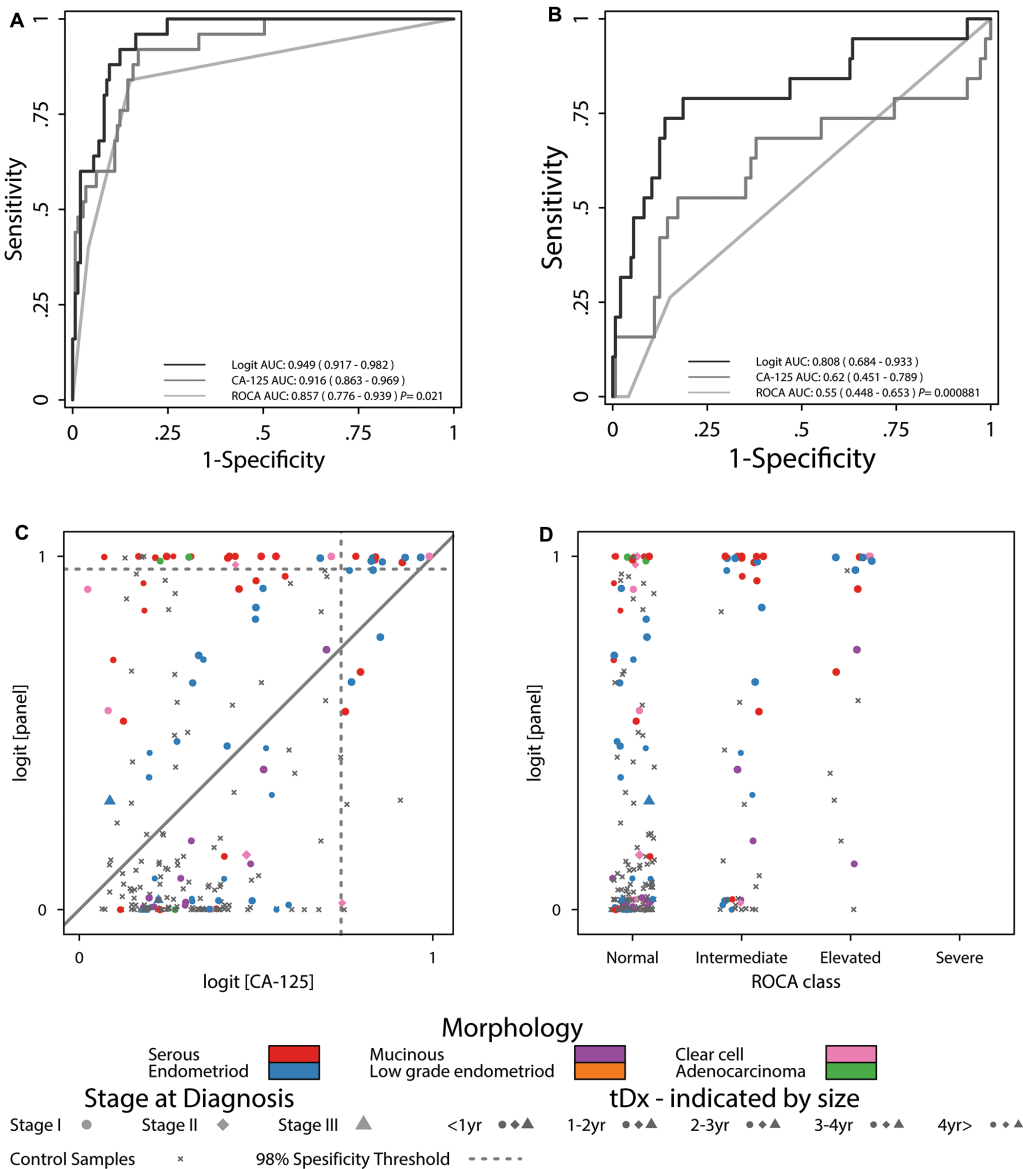
Model I performance compared to CA125 and ROCA (**A**) Type-I ROC curves for less than one year tDx. (**B**) Type-I ROC curves for one to two years tDx. (**C**) Comparisons between Model I scores and logit_CA125_. (**D**) Comparisons between Model I scores and ROCA.

ROC curve analysis on samples (Figure 2) was carried out for Model I, CA125 alone (logit_CA125_) and the ROCA algorithm risk classification, at one year (Figure 2A) and two years pre-diagnosis (Figure 2B) in order to ascertain if the model improved detection in the patient population. For Type I less than one year tDx (Figure 2A) Model I AUC, 0.949 (95% CI 0.917-0.982), was significantly better than the ROCA AUC, which was 0.857 (95% CI 0.776-0.939, *P* = 0.021), and at one to two years tDx (Figure 2B), its AUC of 0.808 (95% CI 0.684–0.933) was significantly better than ROCA with AUC of 0.550 (95% CI 0.448-0.653 *P* < 1 × 10^−3^) In both cases Model I gave a higher AUC than logit_CA125_ alone although in neither case was this significant.

The improvement in risk estimate provided by Model I can be further understood by comparing the risk score for each sample as predicted by Model I against logit_CA125_ (Figure 2C) and ROCA classifications (Figure 2D). The majority (119 of 145) of control samples have lower scores in Model I compared to that for CA125 which confirms the model's superior discriminatory power for the separation of OC cases and control. For Model I a high number of OC cases approaching diagnosis have logit scores near unity (1.0). At 98% specificity, of the 85 Type I samples, Model I (score > 0.96) detected 27 samples from 11 (64%) women, at the same specificity logit_CA125_ (score > 0.74) detected 17 samples from 8 (47%) women (Figure 2C). The cases identified by Model I include 3 endometriod, 5 serous, 2 clear cell and 1 adenocarcinoma whereas the cases identified by logit_CA125_ include 4 endometriod, 2 serous and 2 clear cell. Of these individuals Model I identified 10 diagnosed at stage I and 1 at stage II whereas logit_CA125_ identified 7 diagnosed at stage I and 1 at stage 2. Thus Model I is capable of identifying more OC cases, particularly at stage I, than logit_CA125_.

### Model score lead time comparison

**Figure 3:**
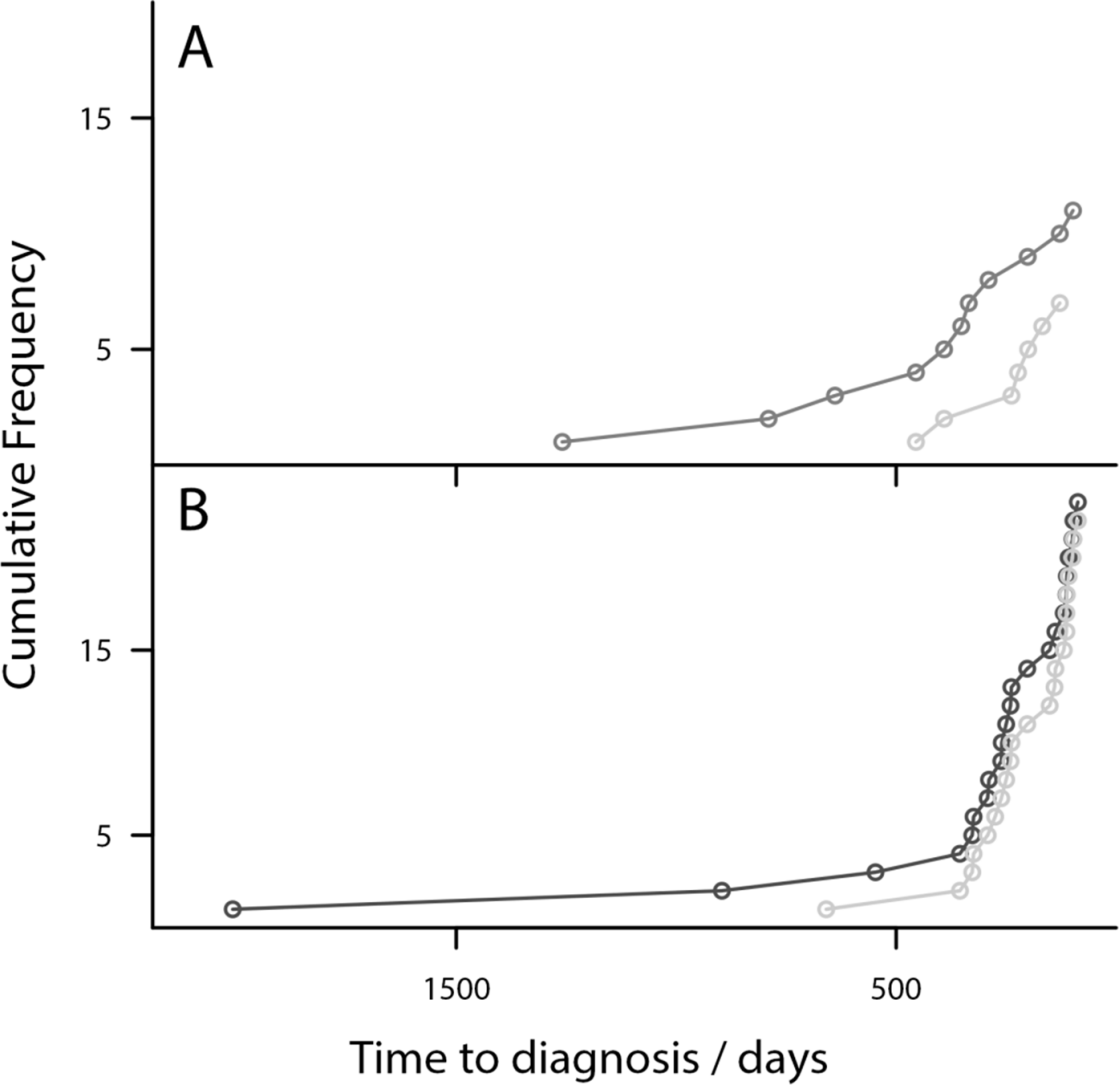
Risk Models detect OC earlier than CA125-Plot showing the cumulative diagnosis of OC cases (**A**) Model I diagnosis of Type I cases (grey) compared to logit_CA125_ (light grey). (**B**) Model II diagnosis of Type II cases (black) compared with logit_CA125_ (light grey). Model I diagnoses cases substantially earlier than logit_CA125_. Model II diagnoses several samples earlier than logit_CA125._

The improvement in lead time achieved by Model I over logit_CA125_ is further illustrated by comparing the cumulative detection of cases over time approaching diagnosis, by finding the earliest in an unbroken chain of OC cases crossing the 98% threshold of the model from the samples closest to diagnosis (Figure 3A). This shows the 11 women with Type I OC are detected substantially earlier than by logit_CA125_.

### Model II outperforms logit CA125 and ROCA

#### Area under the curve analysis

**Figure 4:**
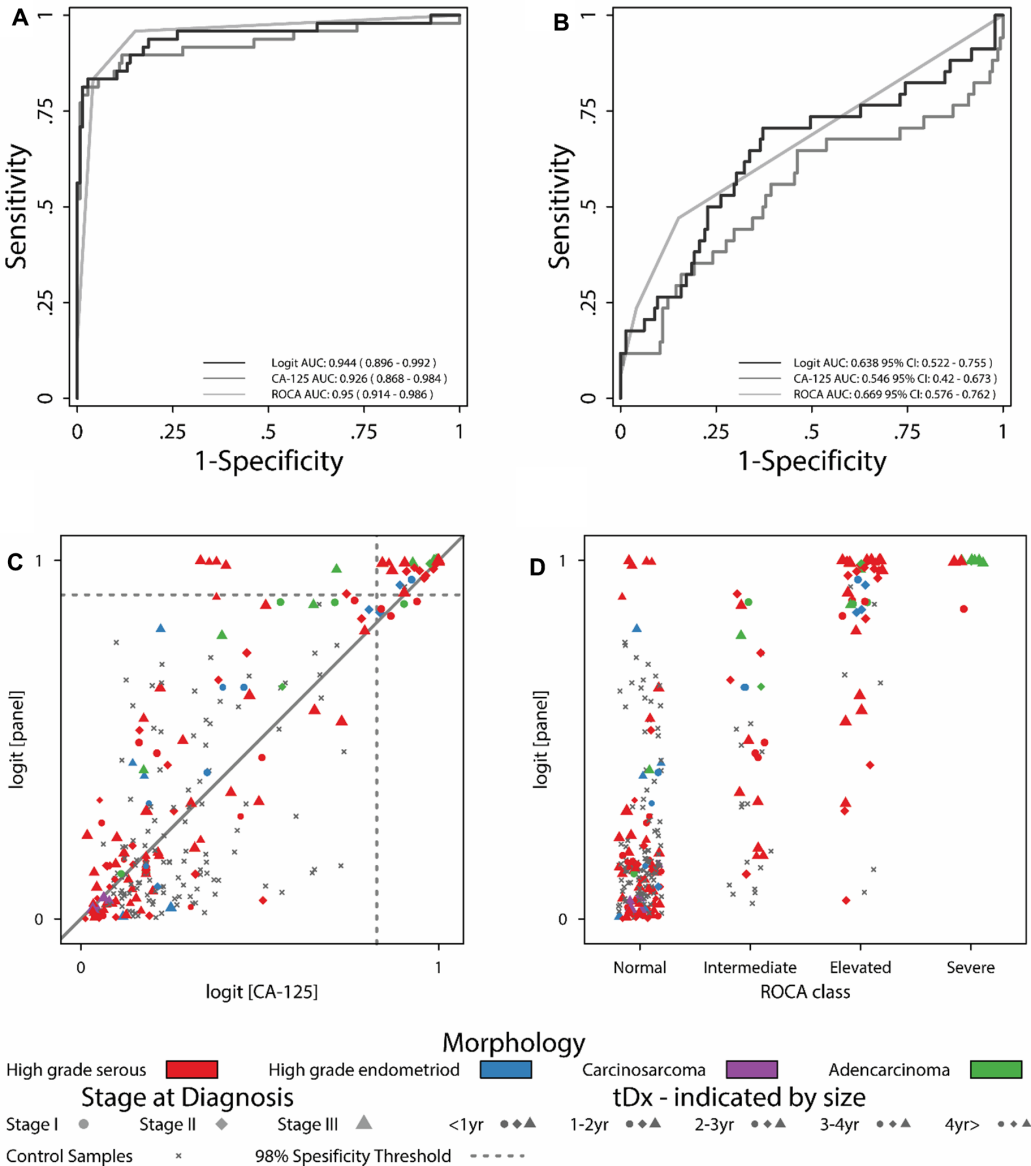
Model II performance compared to CA125 and ROCA (**A**) Type-II ROC curves for less than one year tDx. (**B**) Type-II ROC curves for one to two years tDx. (**C**) Comparisons between Model II scores and logit_CA125_. (**D**) Comparisons between Model II scores and ROCA.

The ROC curve analysis for Type II OC (Figure 4A) demonstrated that Model II was comparable to both logit_CA125_ and ROCA, less than one year tDx with AUCs of 0.944, 0.926 and 0.950 respectively. Similarly for one to two years to diagnosis (Figure 4B) the AUCs were 0.638, 0.546 and 0.669 respectively. As with Model I the majority of controls (99 of 145) are given lower scores by Model II than for CA125 alone which confirms this model's superior discriminatory power for the separation of OC cases and control.

Model II risk estimates are consistently higher than logit_CA125_ risk estimates, demonstrating the models superiority. Within the model scores Type II OC cases (Figure 4C, coloured symbols) were given higher scores than logit_CA125_ particularly as they approach diagnosis. At a specificity of 98% (0.76 logit score), of the 158 Type II samples, Model II detected 54 samples from 24 women (16 with high grade serous, 3 with endometrioid, 3 with adenocarcinoma and 2 with papillary serous cystadenocarcinoma OC). Whereas at the same 98% specificity logit_CA125_ (score > 0.72) detected 43 samples from 22 women (15 with high grade serous, 2 with endometrioid, 3 with adenocarcinoma and 2 with papillary serous cystadenocarcinoma OC).

### Model score lead time comparison

Within the additional 11 samples detected at 98% specificity by Model II, one was an additional high grade serous and endometrioid case diagnosed at Stage III that were previously missed by logit_CA125_. It can also be seen that the 24 women are detected earlier by Model II than by logit_CA125_ (Figure 3B).

Comparison with ROCA (Figure 4D) shows five high grade serous OC samples from a single woman and one high grade endometriod (red and blue symbols) which Model II identified by high scores (Figure 4D) which were previously classified as “normal” by ROCA. Both these cancers were diagnosed at stage III. The endometriod cancer was not diagnosed in the time covered by our sample set (6 years). The serous case was first identified by ROCA 140 days tDx, whereas every single sample in our sample set for this individual crossed the 99th percentile threshold for Model II, from 501 days tDx and as far out as 2008 days tDx. Perhaps representing the detection of a cancer developing slowly through stage I and II over a number of years. This demonstrates the power of Model II to detect OC cases that the ROCA approach either detects later or importantly does not detect.

### Risk models as triage tool for symptomatic women

The models as outlined above are suitable for application in a screening programme context where they require an initial sample to be taken against which velocity of change in protein expression may be calculated.

A potential alternative application of this biomarker panel, once it has undergone further validation, would be as a triage tool for clinicians to assess, from a single blood sample, whether a symptomatic patient is at low or high risk of OC. In this context it may be a useful tool on its own or potentially in combination with current protocols used for differential diagnosis of an adnexal mass in symptomatic patients such as OVA1 [23] or ROMA [10, 24].

Within the current study we cannot directly compare the advantages and disadvantages of our results with ROMA and or indeed OVA1, as stated above both were designed for differential diagnosis of an adnexal mass in symptomatic patients. The UKCTOCS study was not directed at symptomatic high risk populations but rather as a general screening tool.

However, given that OVA1 [23] and ROMA [10, 24] algorithms give significant weighting to CA125 levels, our models can be adapted (as described in methods) and assessed to ascertain, if from the measurement of a single sample, they can improve on CA125 alone.

To test this we combined the models (Supplementary Figure S1) into a schema in which a threshold was set for each model, setting the specificity at > 98% for that model (logit score 0.90 for Model I and 0.77 for Model II), so that should a patient sample exceed the specified threshold on either model, they would be considered at high risk of OC. A follow up sample could then be taken and if the risk was still high they could be sent for further investigations, for example, transvaginal ultrasonography (TVS).

**Figure 5:**
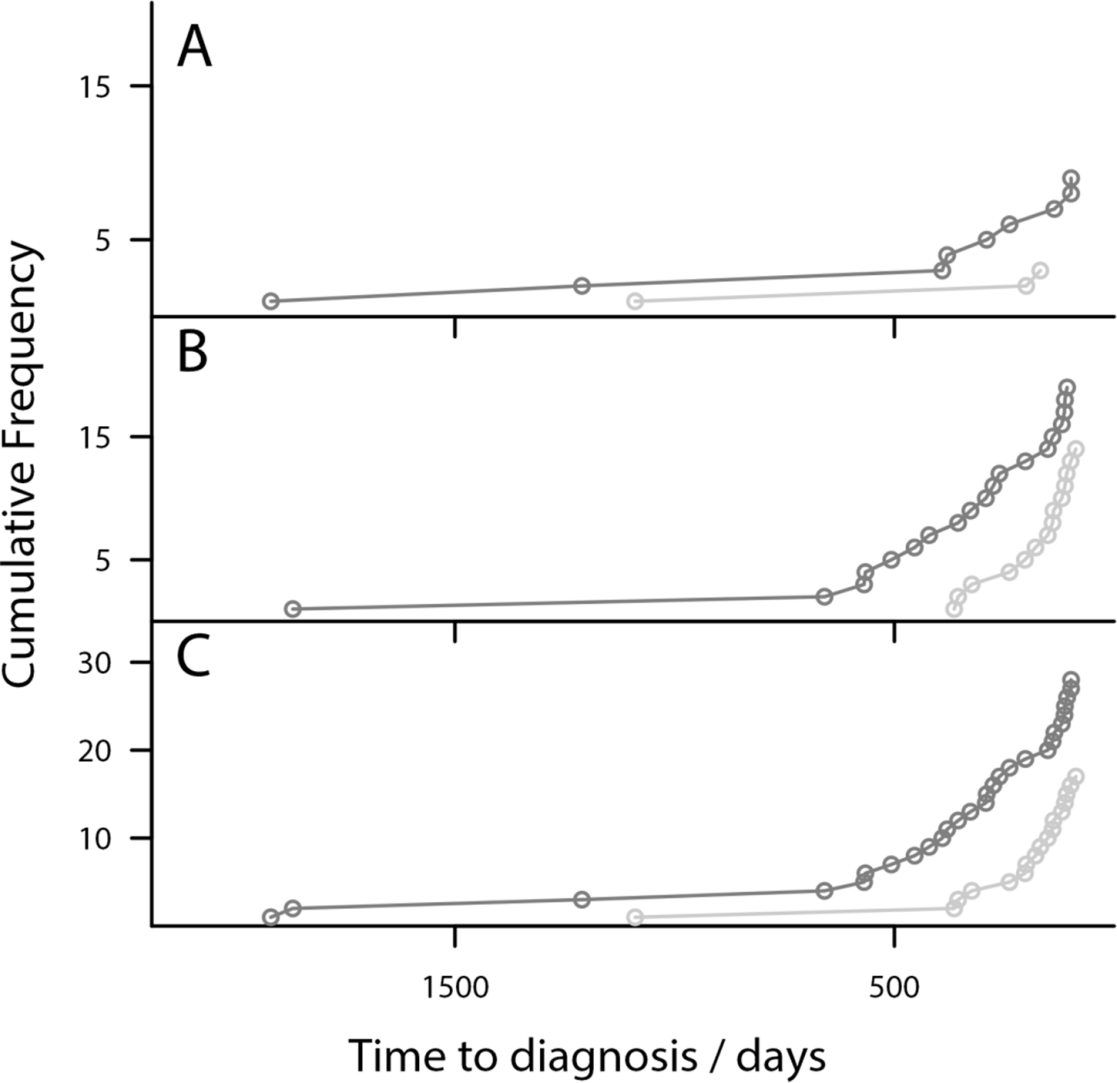
Risk Models detect OC earlier than CA125 in triage algorithm- Plot of the cumulative diagnosis of OC cases using the triage algorithm (**A**) Type I cases only (grey), (**B**) Type II cases only (grey) and (**C**) Combined OC cases (grey), plotted against the CA125 > 35 U/mL clinical threshold (light grey). For Type I cases the algorithm identifies many more samples at much earlier time points. For Type II cases the algorithm also detects OC cases much earlier than the CA125 threshold. The patterns continue for combined OC.

The performance of the triage algorithm (Supplementary Figure S1) was assessed by comparing the earliest in an unbroken chain of OC cases breaching either the Model I or Model II threshold, or the CA125 clinical threshold of 35 U/mL (Figure 5) The results are presented as Type I vs CA125, Type II vs CA125 and as combined ovarian cancer (triage algorithm) vs CA125. In each comparison the model threshold protocol was able to identify OC cases earlier than CA125. The specificity of the protocol determined from all control samples was 95%. The triage tool significantly outperforms CA125 in terms of sensitivity particularly for Type II cases. The sensitivity of the algorithm compared to CA125 at 3, 6, 9 and 12 months tDx are shown in Table 3 for Type I, Type II and combined ovarian cancer (triage algorithm).

**Table 3:** Table of sensitivities obtained for the triage algorithm for Type I, Type II and combined OC cases for the indicated tDx

	Sensitivity Models I and II			Sensitivity logit_CA125_		
Month thx	Type I	Type II	Combined OC	Type I	Type II	Combined OC
< 3	0.53	0.83	0.72	0.53	0.53	0.53
3–6	0.53	0.6	0.57	0.47	0.3	0.34
6–9	0.41	0.53	0.45	0.24	0.2	0.21
9–12	0.3	0.33	0.3	0.24	0.13	0.17

## DISCUSSION

Using serial samples from UKCTOCS, we developed two risk models that demonstrate superior clinical performance for OC risk prediction over CA125 alone and ROCA. Leave-one-out analysis of models indicates the models are robust to sample exclusion and not over fitted to the dataset. Between them Models I and II incorporate four proteins: CA125, Protein Z, Fibronectin and C-reactive protein, and interaction terms between Protein Z and fibronectin with age. Of the four proteins CA125 is the current best biomarker used in clinical practise [6–11]. C-reactive protein is also known to be associated with ovarian cancer [18, 19] as well as having a general association with injury and infectious disease [25]. The role of Fibronectin and Protein Z in ovarian cancer is less well defined. Fibronectin provides a substrate in the extracellular matrix for cell adhesion and migration [26] playing a critical role in healthy development and wound healing. It has been found in elevated concentrations in the vicinity of tumours including those of breast [27] and ovarian [28] cancer where its elevation indicates poorer prognosis, presumably because it facilitates cancer cell invasion of surrounding tissues [29].

Protein Z is part of the haemostatic system in which it supresses clot formation by deactivating factor X in combination with its partner protein Z protease inhibitor [30]. Haemostatic system inhibitors are known to play a role in determining the malignancy of cancers [31]. Protein Z has previously been found down regulated in patients diagnosed with acute leukaemia compared with controls [32], but has also been found to be upregulated in tissues of gastric [33], breast [34] and colon [35] cancers. It is possible the down regulation of serum Protein Z reflects sequestration to the tumour site where it may prevent coagulation.

ROC, area under the curve, analysis confirms that Model I outperforms CA125 and ROCA in both specificity and sensitivity. Model I returns higher risk scores for OC cases and importantly it is able to identify some cases that either CA125 or ROCA detected later, or not at all. Lowess analysis demonstrates that the inflection point at which scores for women with Type I tumours start to rise is approximately 1000 days (~3 years) tDx, compared to 400 days (~1 year) tDx for CA125 alone. Furthermore, the lowess trend for control subject scores does not rise. Model I can discriminate between OC cases and controls, and it does so earlier in the disease process than CA125 alone. The Type I model had better AUC than both ROCA and logit_CA125_, offering the potential to detect Type I OC cases with greater sensitivity and specificity than is currently possible, even one to two years tDx. When considering lead time, women enrolled in UKCTOCS were likely to be detected on average 1–2 years before they would have been clinically. Therefore, Model I potentially offers a lead time of 3 to 4 years for Type I OC.

Model II outperforms both CA125 and ROCA for the detection of Type II OC. Whilst the ROC curves suggest similar performance to a model based on CA125 alone (logit_CA125_) and ROCA, crucially Model II returns higher risk scores for OC cases and importantly it is able to identify some cases that either CA125 or ROCA detected later. Comparisons of Model II and logit_CA125_ scores show Model II assigns higher scores, and is therefore more confident in its identification of a set of 54 samples from 24 women (16 with high grade serous, 3 with endometrioid, 3 with adenocarcinoma and 2 with papillary serous cystadenocarcinoma OC) with 6 of these diagnosed at stage I and 7 at stage II.

Cumulative diagnostic plots show Model II consistently identifies cases earlier than logit_CA125_. Lowess analysis demonstrates that the inflection point where scores for women with Type II tumours start to rise is approximately 800 days (~2 years) tDx, compared to 400 days (~1 year) tDx for CA125. Furthermore, the lowess trend for control subject scores shows near flat expression. Significantly, Model II can discriminate between OC cases and controls and it does so earlier in the disease process than CA125 alone and therefore potentially offers a lead time of 2 to 3 years pre-diagnosis.

The comparison with ROCA is more problematic as the OC cases in the UKCTOCS study were identified by this method, which means that the sample population almost certainly overestimates ROCA's performance in a general population. This is substantiated by the greater ROCA AUC observed at less than one year prior to diagnosis for our sample set 0.950 (95% CI: 0.914–0.986) compared with that of the entire UKCTOCS trial dataset 0.915 (95% CI: 0.883-0.946). Despite this, a similar discrimination is seen for the Type II cases when comparing Model II with ROCA as was seen for the comparison with CA125 alone.

For Model II, 6 OC samples, 5 high grade serous (serial samples from a single case) and one endometriod, were classified as ‘normal’ by ROCA. However, these cases were given high scores by Model II indicating that for some aggressive OC cases Model II has the potential to detect them earlier than ROCA. Within Model II a small number of control samples were assigned elevated scores. This would not be problematic in a screening context, as one can see from the serial analysis of these control samples that their levels return to normal after a single elevated score. However, for OC patients once there is an increase in the score this continues to rise in subsequent serial samples. This would be accommodated in a screening protocol by having a follow up confirmatory sample.

Both Models demonstrate the potential for use as triage tools for women presenting with symptoms of OC or a pelvic mass (Supplementary Figure S1). The models could be used either on their own or in combination with established algorithms such as OVA1 or ROMA. A high risk score on either model would result in a follow up sample being taken if this was still high then further investigation for suspected OC should follow; a low risk score would encourage the clinician to consider alternative diagnoses.

We have developed two risk models based on panels of serum biomarkers that outperform CA125 in the detection of both Type I and Type II OC. Model II identifies the biologically aggressive Type II OC at an earlier stage than CA125 alone, offering the potential for earlier diagnosis and improved survival for women with this poor prognosis disease. The panels proposed here, whilst impressive, require further validation in a larger, independent sample set, containing samples from patients with benign ovarian tumours and other diseases in order to add confidence in their utility as screening tools. However, in the current context they add significantly to the knowledge in the field of early detection biomarkers for OC and move us closer to the identification of biomarker panels and risk models for the early detection and diagnosis of OC.

## MATERIALS AND METHODS

### Study population

This study analysed 418 prospectively collected serial serum samples from women in the UKCTOCS [9, 15, 16] (ISRCTN22488978; NCT00058032), a randomised control trial designed to assess the effect of screening on mortality [15]. Over 202,000 women from 13 centres across the UK were recruited and separated into three cohorts for investigation. The research presented here used serum samples collected from women in the multimodal screening arm of UKCTOCS, which consisted of > 50,000 women who had serial serum samples taken over a ten year period. UKCTOCS also collected detailed epidemiological data from health questionnaires and clinical data for OC cases including tumour classification, morphology and stage at diagnosis.

The serum set investigated here comprised eighty women from the multimodal group of UKCTOCS; 49 diagnosed with invasive epithelial (9 Type I and 30 Type II) and borderline (10) (as with previous studies we grouped borderline tumours with Type I for analysis) [17, 36–38] and thirty one controls matched to the Type II OC cases by age, collection centre and date. A study design flowchart is presented for this cohort in Supplementary Figure S2 and information on baseline characteristics can be found in Table 4 and Supplementary Table S1 [17]. This set contained all of the invasive ovarian cancer samples available that had serial samples spanning less than 14 months to diagnosis right through to greater than 32 months and up to 72 months to diagnosis. Two control women were excluded because they went on to develop either skin or breast cancer. One Type I sample was excluded because only two samples were available so that only a single sample was available after scaling to the first sample. A second Type I subject was removed; as the single example of Papillary serous cystadenoma borderline malignancy in the sample set it was diagnosed at stage Ia and did not display any elevation in CA125 and therefore effectively behaved as a control over the time window of analysis.

**Table 4:** Information on the OC sample population used within the study including, type, morphology and stage at diagnosis

	Number			
Cancer Type	All	StageI	StageII	StageIII
**Type I**	19	16	2	1
**Borderline**	10	10	.	.
Serous	6	6	.	.
Mucinous	2	2	.	.
Endometrioid	2	2	.	.
**Invasive**	9	6	2	1
Low grade endometrioid	5	3	1	1
Clear cell	3	2	1	.
Adenocarcinoma	1	1	.	.
**Type II**	30	7	8	15
High grade serous	23	5	6	12
High grade endometrioid	3	1	1	1
Carcinosarcoma	1	.	.	1
Adencarcinoma	3	1	1	1

### ELISA assays

Candidate biomarkers: Fibronectin and C-reactive protein (uniprot accession numbers: P02751 and P02741 respectively) were quantified in all 418 serum samples by ELISA kit assays (Abcam, Cambridge, UK) according to manufacturer's instructions, duplicate readings, average CV's were 5% and 4.3% respectively. Protein Z serum values (uniprot accession numbers: P22891) for all samples were available from a study we recently carried out [17]. Serum CA125 levels were available as part of UKCTOCS for the set as previously described [9].

### ROCA classification

CA125 results within UKCTOCS were interpreted using the ROCA algorithm [11, 39]. All women randomised to the multimodal group of the trial underwent annual CA125 testing and based on the ROCA classification, they were either returned to annual screening (if their risk was normal) or triaged to repeat CA125 testing in three months (if intermediate risk) or repeat CA125 and transvaginal ultrasound in six weeks (if elevated risk) [9, 15, 16]. Those with persistently elevated risk were sent for clinical assessment with a view to surgery. The trial protocol is detailed elsewhere [9, 15, 16].

### Data analysis

Data was processed, analysed, and figures produced using the R (3.2.4) statistical environment (www.r-project.org), all *P* values are two-sided. All protein abundances were normalised to that of the earliest collected sample (as with established biomarker longitudinal analysis (ROCA [8]), velocity rather than absolute levels of biomarkers were the basis of models), log transformed and scaled to unit variance. In order to keep variables at comparable magnitudes the age of the women at sample collection was rescaled by subtracting the median age in the data set. The controls were matched to the Type II OC cases by age, collection centre and date. As controls do not have a time to diagnosis, for comparison with cases, samples from the control women were allocated a tDx value so that the last sample for each control was matched to the last sample from the matched case.

Logit regression returns the probability that a given subject is a case rather than a control. Here separate multivariable logistic regression models for Type I and Type II OC cases against the shared controls were built to assess the additional contribution candidate biomarkers might make as part of a diagnostic panel.

For each OC type a logit model was trained on OC cases less than 400 days to diagnosis, (chosen as this was the point at which CA125 began to elevate in OC cases), against matched controls, by analysis of the levels of the putative biomarker panel and selected interactions between age and panel members.

The models were assessed for overfitting using established methodologies for sample sets of this size in ovarian cancer research [20–22], by leave-one-out cross validation. This confirmed the stability of the model's coefficients and the logit odds of the excluded samples.

Models were compared with nested alternatives by likelihood ratio tests of the residual variance with the chi-squared test. The improved descriptive power of the putative panel over CA125 alone was confirmed by eliminating all terms bar CA125 from Models I and II. The possibility that the epidemiological factors such as body mass index (BMI), hormone replacement therapy (HRT) or the contraceptive pill affected the model was excluded by training an expanded version of each of model I and model II which included these epidemiological factors.

Predictive power of the biomarker panel models in terms of specificity and sensitivity was compared with ROC curve analysis against the UKCTOCS ROCA classification and prediction based on CA125 levels alone using the pROC package in R [40] for the time periods 0–1 years tDx and 1–2 years tDx.

A ‘potential future’ alternative application of this biomarker panel may be as a triage tool for clinicians to assess, from a single blood sample, whether a symptomatic patient is at low or high risk of OC. The models outlined above were adapted for this purpose, by normalising the expression of each protein to the median level observed in the controls, rather than the initial sample taken from a subject, producing a model applicable to a single sample, such as would be obtained in primary care. The models were again trained as outlined above.

Cumulative diagnostic plots were created by plotting accumulated diagnosis against tDx. In order to exclude false positive diagnosis that might inflate the diagnosis rate, an unbroken chain of positive diagnosis were required between a positive diagnosis and the last two samples acquired prior to diagnosis.

## SUPPLEMENTARY MATERIALS


