# Supplementary material for: Novel risk models for early detection and screening of ovarian cancer
Source: Oncotarget. 2016 Nov 26;8(1):785–97. doi: 10.18632/oncotarget.13648 (PMC5352196; doi:10.18632/oncotarget.13648)
Supplement: Supplementary file 1 [file oncotarget-08-785-s001.pdf]

Novel risk models for early detection and screening of ovarian cancer

Supplementary Materials

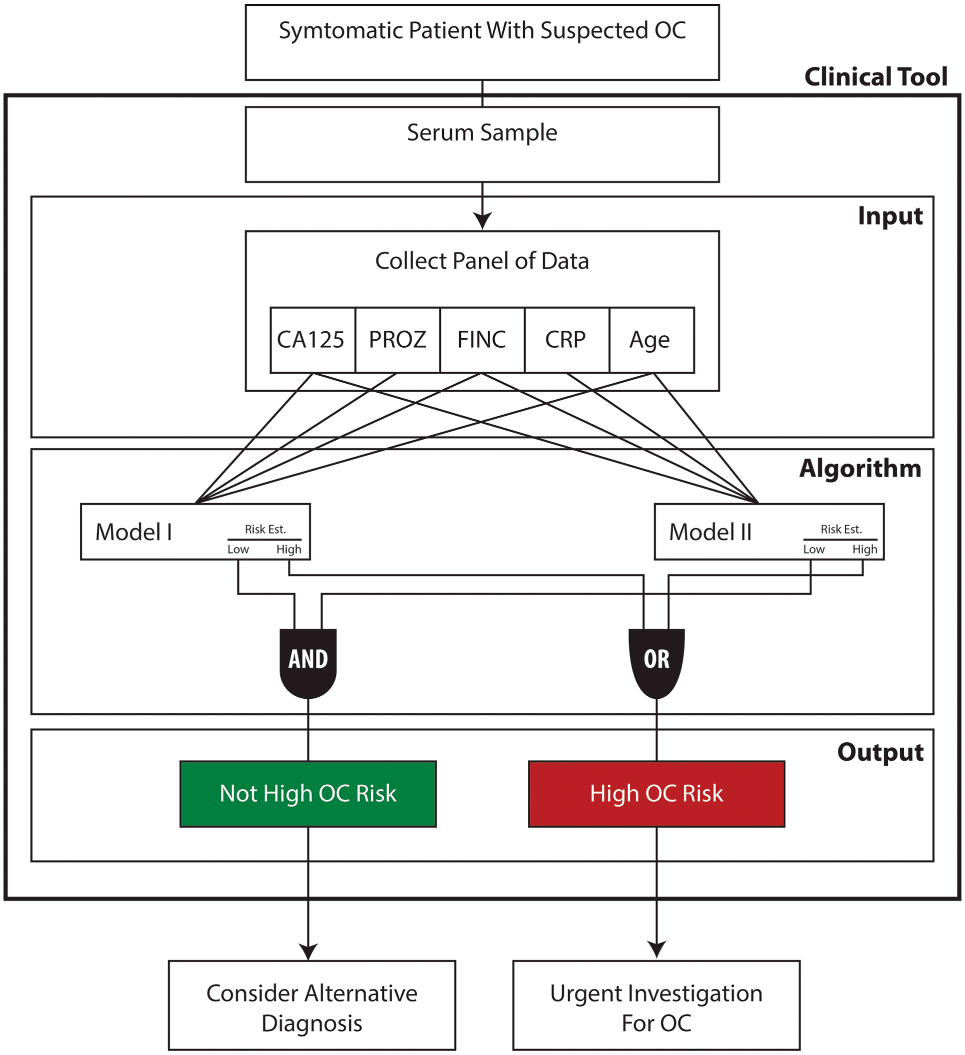

Supplementary Figure S1: Schematic diagram of possible implementation of the risk models in clinical practice. Symptomatic patients would have blood sampled and every member of the protein panel quantified. If either model returned a high risk result the patient would have a follow up sample taken, if this confirmed high risk they would be referred urgently for further investigations. If neither model returned high risk the patient would be investigated for other causes of their symptoms

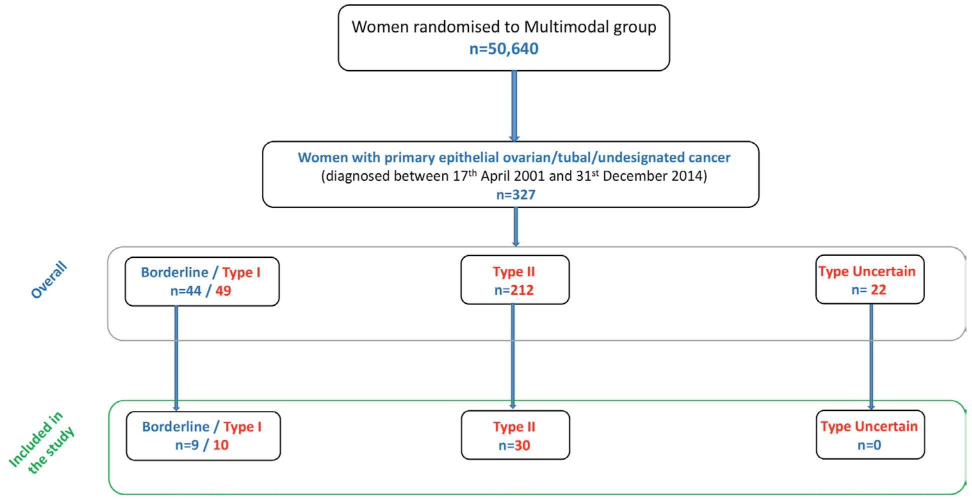

Supplementary Figure S2: Workflow summary of the origin of the samples used in this study.

Supplementary Table S1: Baseline characteristics of UKCTOCS participants

	Median (25th - 75th centiles)							
	Control		Ovarian cancer					
			Overall		Type I		Type II	
	***n* = 31**		***n* = 49**		***n* =19**		***n* = 30**	
**Age (years) at randomisation**	60.8 (58.4–65.8)		62.8 (58.7–67.3)		64.2 (58.9–69.9)		61.1 (58.7–65.5)	
**Years since last period at randomisation**	12.6 (6.6–18.2)		11.4 (5.7–18.2)		15.2 (8.1–22.6)		10.7 (4.0–16.1)	
**Duration of HRT use in those who were on HRT at randomisation (yrs)**	6.9 (5.8–11.7)		9.7 (4.8–13.0)		13.0 (10.7–13.9)		7.2 (3.3–11.6)	
**Duration of OCP use (yrs) in those who had used it**	10 (3–12)		6 (3–8)		5 (3–8)		6 (4–8)	
**Miscarriages (pregnancies < 6mths)**	0 (0–1)		0 (0–0)		0 (0–1)		0 (0–0)	
**No. of children (pregnancies > 6mths)**	2 (0–2)		2 (2–2)		2 (1–2)		2 (2–3)	
**Height (cms)**	162.6 (158.8–167.6)		162.6 (157.5–165.1)		162.6 (157.5–166.4)		162.6 (157.5–165.1)	
**Weight (kg)**	65.3 (62.6–74.0)		69.9 (62.6–78.9)		71.2 (66.7–79.2)		65.9 (61.7–75.8)	
	**Number (%)**							
	**No.**	**%**	**No.**	**%**	**No.**	**%**	**No.**	**%**
**Ethnicity:**								
**White**	30	96.8%	48	98.0%	18	94.7%	30	100.0%
**Black**	0	0.0%	0	0.0%	0	0.0%	0	0.0%
**Asian**	0	0.0%	0	0.0%	0	0.0%	0	0.0%
**Other**	1	3.2%	1	2.0%	1	5.3%	0	0.0%
**Missing**	0	0.0%	0	0.0%	0	0.0%	0	0.0%
**Hysterectomy**	1	3.2%	6	12.2%	2	10.5%	4	13.3%
**Ever use of oral contraceptive pill**	18	58.1%	21	42.9%	9	47.4%	12	40.0%
**Use of HRT at recruitment**	8	25.8%	9	18.4%	3	15.8%	6	20.0%
**Personal history of cancer***	0	0.0%	2	4.1%	1	5.3%	1	3.3%
**Personal history of breast cancer**	0	0.0%	1	2.0%	1	5.3%	0	0.0%
**Maternal history of ovarian cancer**	0	0.0%	1	2.0%	0	0.0%	1	3.3%
**Maternal history of breast cancer**	4	12.9%	4	8.2%	1	5.3%	3	10.0%

* includes those with personal history of breast cancer
